# Supplementary material for: Dysbiosis of fecal virome in pediatric Crohn’s disease and its dynamic changes during infliximab therapy
Source: mSystems. 2026 Mar 9;11(4):e01489-25. doi: 10.1128/msystems.01489-25 (PMC13098212; doi:10.1128/msystems.01489-25)
Supplement: Supplemental file — Table S1; Fig. S1 and S2. [file msystems.01489-25-s0001.docx]

**Supplementary TABLE 1** Clinical characteristics of the study cohort between the baseline levels of remission and non-remission groups.

|  | Remission (n=41) | Non-remission (n=12) | P value |
| --- | --- | --- | --- |
| Age, years | 12.9 (11.9，13.9) | 12.0 (10.3，13.7) | 0.371 |
| Male, n (%) | 27 (66.8) | 3 (25.0) | 0.034 |
| BMI, Kg/m^2^ | 15.1 (13.9，16.9) | 14.0 (12.8，15.3) | 0.001 |
| **Location** |  |  |  |
| L1，n (%) | 3 (7.3) | 0 (0) |  |
| L2，n (%) | 1 (2.4) | 1 (8.3) |  |
| L3，n (%) | 35 (85.4) | 10 (83.4) |  |
| L4，n (%) | 2 (4.9) | 1 (8.3) |  |
| **Clinical index** |  |  |  |
| WBC (*10^9/L) | 9.6 (7.4，11.6) | 8.2 (6.9， 12.8) | 0.664 |
| PLT (*10^9/L) | 475.0 (363.0，567.0) | 435.0 (331.3， 546.5) | 0.941 |
| CRP (mg/L) | 32.0 (14.5，49.0) | 37.5  (7.5，72.5) | 0.941 |
| ESR (mm/h) | 86.0 (47.0，108) | 88.5 (44.8， 120.0) | 0.941 |
| Hb (g/L) | 106.0 (98.5，118.5) | 98.5 (86.5，120) | 0.275 |
| PCDAI | 32.5 (26.0，42.5) | 31.3 (13.0，45.9) | 0.775 |
| FCP (ug/g) | 450.0 (162.2，450.0) | 419.6 (120.3，817.4) | 0.399 |

BMI, Body Mass Index; WBC, white blood cells; PLT, platelets; CRP, C-reactive protein; ESR, erythrocyte sedimentation rate; Hb, hemoglobin; PCDAI, Pediatric Crohn’s Disease Activity Index; FCP, fecal calprotectin.

The data were analyzed using the nonparametric Wilcoxon rank-sum test (two groups).


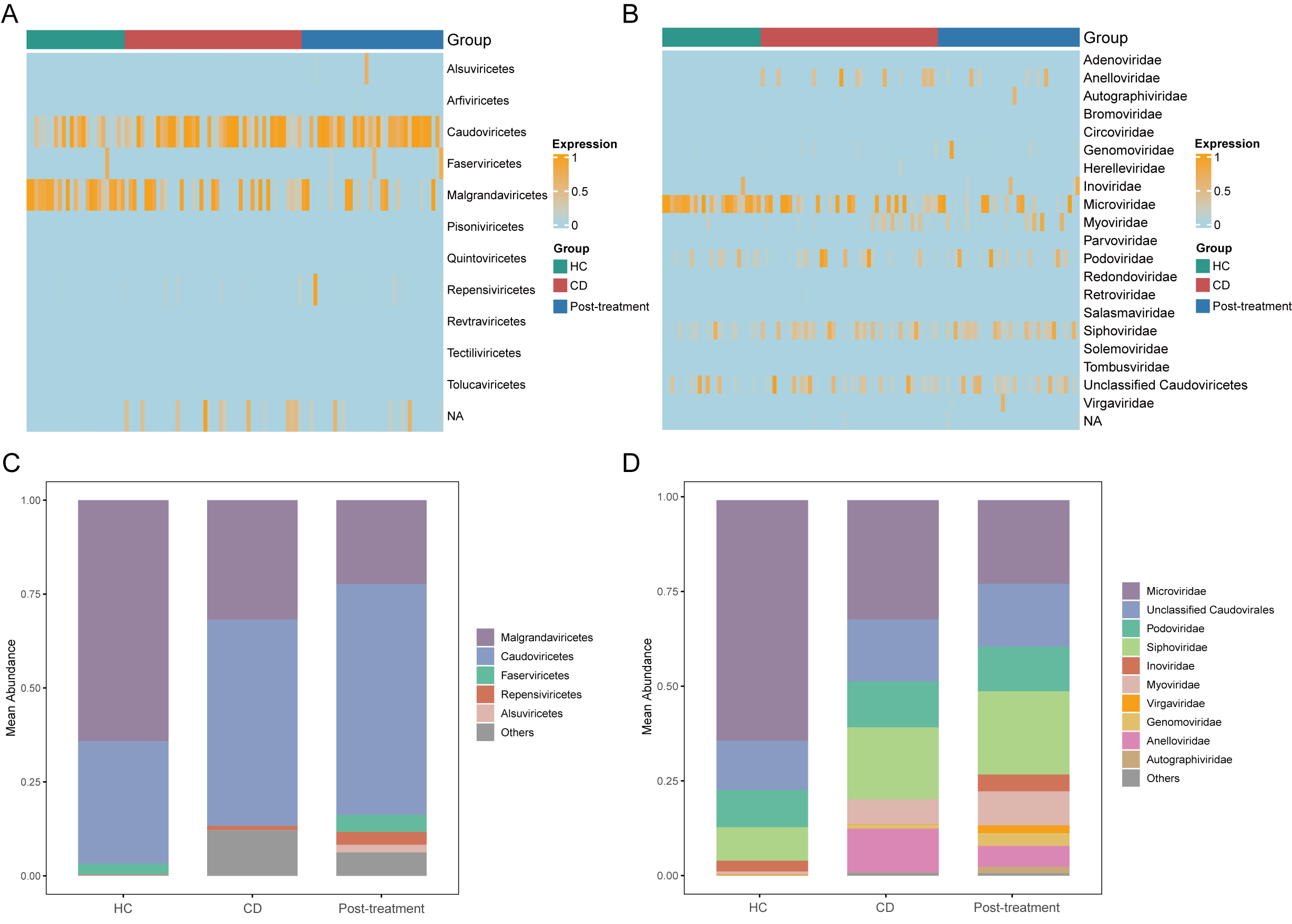


**Supplementary Fig 1** Overview of the fecal virome in all fecal samples. Heatmap showing viral profiles in pediatric Crohn's disease (CD), healthy controls (HC) and pediatric CD patients who have received IFX treatment (Post-treatment) at class level (A) and family level (C). Boxplots showing the different viral at class level (B) and family level (D).


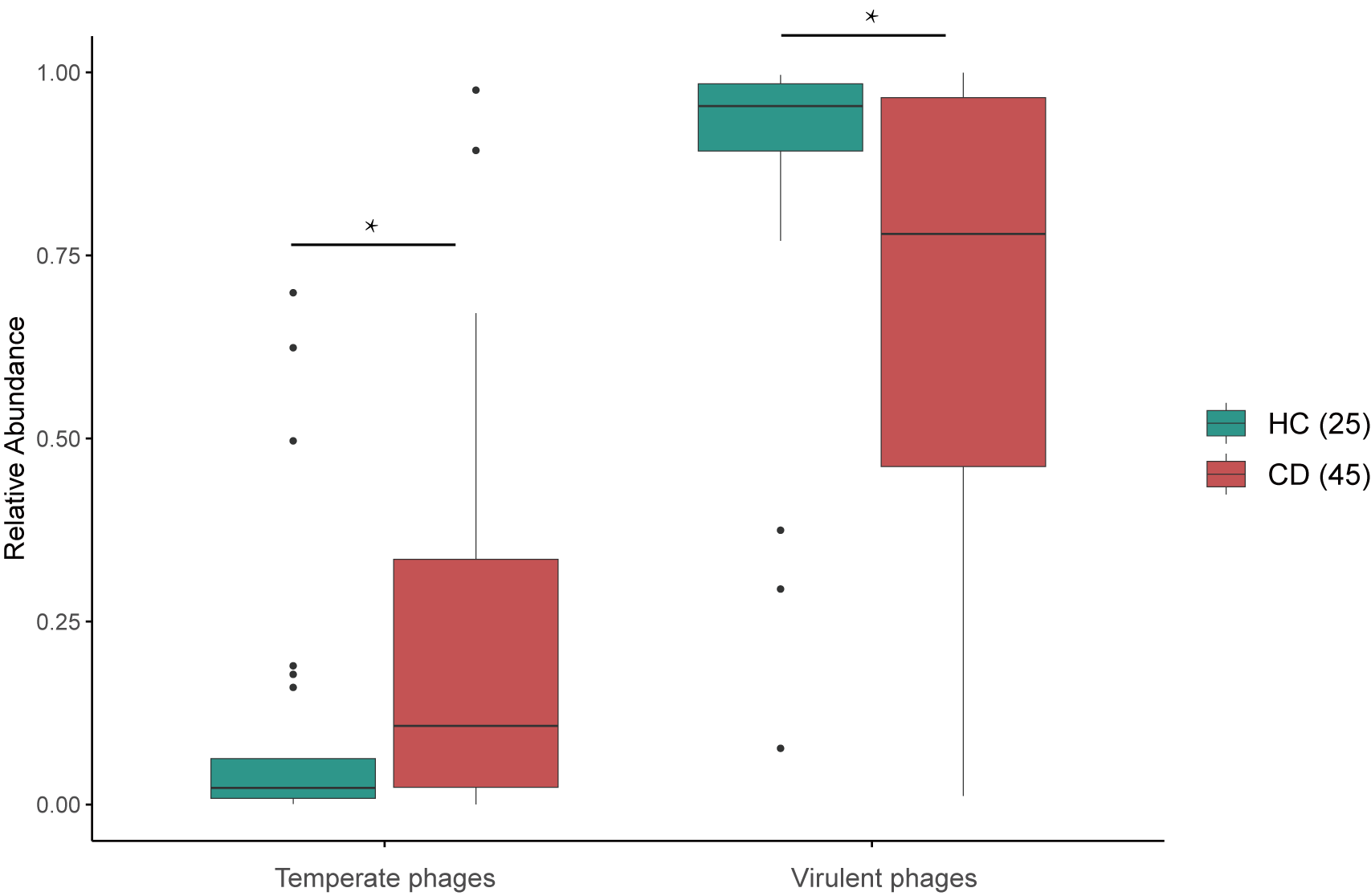


**Supplementary Fig 2** Relative abundance of temperate phages and virulent phages between pediatric Crohn's disease (CD) and healthy controls (HC).
